# Hemodynamic Responses in Carotid Bifurcation Induced by Enhanced External Counterpulsation Stimulation in Healthy Controls and Patients With Neurological Disorders

**DOI:** 10.3389/fphys.2021.717080

**Published:** 2021-08-31

**Authors:** Shuai Tian, Wei Pan, Junping Peng, Hui Wang, Bin Deng, Yi Liang, Xinghua Li, Huahui Liu, Yujia Wang, Bin Luo, Jianhang Du

**Affiliations:** ^1^Department of Cardiology, The Eighth Affiliated Hospital of Sun Yat-sen University, Shenzhen, China; ^2^Guangdong Innovative Engineering and Technology Research Center for Assisted Circulation, Sun Yat-sen University, Shenzhen, China; ^3^Department of Radiology, The Eighth Affiliated Hospital, Sun Yat-sen University, Shenzhen, China; ^4^Department of Ultrasound, The Eighth Affiliated Hospital, Sun Yat-sen University, Shenzhen, China; ^5^Medical Imaging Center, Chongming Medical Technology Company, Shenzhen, China; ^6^Department of Neurosurgery, The Eighth Affiliated Hospital, Sun Yat-sen University, Shenzhen, China; ^7^National Health Commission Key Laboratory of Assisted Circulation, Sun Yat-sen University, Guangzhou, China

**Keywords:** carotid bifurcation, blood flow distribution, cerebral autoregulation, enhanced external counterpulsation, wall shear stress

## Abstract

Enhanced external counterpulsation is a Food and Drug Administration–approved, non-invasive, assisted circulation therapy for ischemic cardiovascular and cerebrovascular diseases. Previous studies have confirmed that EECP stimulation induces largely different cerebral hemodynamic responses in patients with ischemic stroke and healthy controls. However, the underlying mechanisms remain uncertain. We hypothesize that different blood redistributions at the carotid bifurcation may play a key role. Ten subjects were enrolled in this study, namely, five patients with neurological disorders and five young healthy volunteers as controls. Magnetic resonance angiography (MRA) was performed on the carotid artery. All the subjects received a single session of EECP treatment, with external cuff pressures ranging from 20 to 40 kPa. Vascular ultrasound measurements were taken in the common carotid artery (CCA), external carotid artery (ECA) and internal carotid artery (ICA). Three-dimensional patient-specific numerical models were developed to calculate the WSS-derived hemodynamic factors. The results indicated that EECP increased CCA and ECA blood flow in both groups. The ICA blood flow in the patient group exhibited a mean increase of 6.67% during EECP treatment compared with the pre-EECP condition; a mean decrease of 9.2% was observed in the healthy control group. EECP increased the averaged wall shear stress (AWSS) throughout the carotid bifurcation in the patient group; the ICA AWSS of the healthy group decreased during EECP. In both groups, the oscillatory shear index (OSI) in the ICA increased proportionally with external cuff pressure. In addition, the relative resident time (RRT) was constant or slightly decreased in the CCA and ECA in both groups but increased in the ICA. We suggest that the benefits of EECP to patients with neurological disorders may partly result from blood flow promotion in the ICA and increase in WSS at the carotid bifurcation. In the healthy subjects, the ICA blood flow remained constant during EECP, although the CCA blood flow increased significantly. A relatively low external cuff pressure (20 kPa) is recommended as the optimal treatment pressure for better hemodynamic effects. This study may play an important role in the translation of physiological benefits of EECP treatment in populations with or without neurological disorders.

## Introduction

Enhanced external counterpulsation is an FDA-approved atraumatic therapy that uses a non-invasive circulatory support device to improve blood perfusion in ischemic organs such as the heart and brain (Werner et al., 1999; Michaels et al., 2002; Lin S. et al., 2012). Enhanced external counterpulsation (EECP) treatment involves the application of three sets of pneumatic cuffs wrapped around the calves, lower thighs, and upper thighs of a patient, with sequential inflations and deflations synchronized with electrocardiogram (Zheng et al., 1984). In clinical applications, EECP usually uses a treatment pressure (external cuff pressure) ranging from 30–35 kPa, and it may lead to several acute hemodynamic effects, namely, (I) diastolic augmentation and systolic unloading, which are similar to those of intra-aortic balloon pump (IABP) treatment (Taguchi et al., 2000; Michaels et al., 2002); (II) increase in cardiac output and oxygen uptake (Ahlbom et al., 2016); (III) venous return augmentation and afterload reduction (Lawson et al., 2004); (IV) improvement in left ventricular hemodynamics (Eftekhari and May, 2012).

For years, enhanced external counterpulsation has been used in the treatment of ischemic cerebrovascular diseases (Han and Wong, 2008; Han et al., 2008; Jauch et al., 2013; Liu et al., 2018), and it has been given a Class IIa recommendation in the Guideline for the Early Management of Patients with Acute Ischemic Stroke by the American Stroke Association (ASA) (Jauch et al., 2013). Liu et al. (2018) reported that 10 daily sessions of EECP can enhance ipsilesional corticomotor excitability and reduce motor impairment of the paretic hand in patients who experienced a subacute stroke. Han et al. (2008) reported that a 7-week course of EECP treatment (35 h) could lead to a significant overall improvement in the National Institutes of Health Stroke Scale (NIHSS) rating and color velocity imaging quantification (CVIQ) for patients with ischemic stroke. Tian et al. (2016) reported that long-term EECP treatment could improve the clinical outcome of patients with stroke by decreasing the beat-to-beat BPV.

It is suggested that for cerebral vascular disease, changes in cerebral blood flow velocity (CBFV) and perfusion during the treatment contribute to the clinical benefits of EECP (Han et al., 2008). However, the underlying hemodynamic mechanism remains uncertain and controversial. Measurements in human subjects and animal models have demonstrated a significant increase in blood flow in the common carotid artery (CCA) during EECP (Applebaum et al., 1997; Werner et al., 1999; Levenson et al., 2007; Zhang et al., 2010). Werner et al. (1999) reported a 19 and 26% blood flow volume increase during EECP in internal carotid arteries (ICAs) of healthy volunteers with 200 and 300 mmHg cuff pressure, respectively. The same research group further reported that EECP with a cuff pressure of 250 mmHg led to a plateau or relative decrease in blood flow velocities in the middle cerebral artery (MCA) both in healthy controls and patients with severe coronary atherosclerosis (Werner et al., 2003). Werner et al. (2004) observed a significant perfusion increase in the ischemic retina area of central retinal artery occlusion (CRAO) and branch retinal artery occlusion (BRAO). Jungehuelsing et al. (2010) and Lin et al. (2014) confirmed the plateau of mean CBFV in young and elderly healthy subjects during EECP treatment, although mean blood pressure and peak diastolic CBFV increased significantly. However, Lin W. H. et al. (2012) observed a significant increase in mean CBFV and cerebral blood flow in ischemic stroke patients during EECP treatment. In later research, Lin et al. (2014) reported a plateau or relative decrease in CBFV following an increase in EECP cuff pressure from 150 to 262.5 mmHg; they recommended rather low 150 mmHg as the optimal treatment pressure for ischemic stroke. It has been suggested that cerebral autoregulation (CA) and impaired CA play key roles in modulating cerebral blood flow during EECP (Werner et al., 2003; Lin W. H. et al., 2012; Lin et al., 2014), similar to dynamic physical exercise (Querido and Sheel, 2007; Sato et al., 2011; Chen et al., 2020). We hypothesize that the influence of EECP on cerebral circulation may be partly derived from the redistribution of blood flow in the carotid bifurcation, and is different on healthy subjects and patients with neurological disorders.

The blood flow promotion of EECP produces variations in the arterial biomechanical environment. Biomechanical factors, especially wall shear stress (WSS), are believed to play a key role in maintaining the physiological functions of arteries, and in pathological changes (Bonetti et al., 2003; Casey et al., 2008). Thus, long-term EECP was suggested for its potential vasculoprotective and anti-atherosclerotic effects through improved WSS in clinical observations and animal experiments (Zhang et al., 2007, 2010; Braith et al., 2010). However, the influence of EECP on WSS-derived hemodynamic factors remains unclear. Xu et al. (2020) reported that EECP treatment reduced the surface area ratio of the time-averaged wall shear stress risk area (SAR–TAWSS) and the oscillatory shear index risk area (SAR–OSI) in coronary arteries of patients who had undergone stent implantation or coronary artery bypass grafting.

In the previous study (Du and Wang, 2015), we confirmed the influence of EECP on CCA blood flow pattern and perfusion based on invasive measurements in a porcine model. We proposed a high-precision, patient-specific numerical scheme to assess the impact of EECP on WSS-related factors in the carotid bifurcation (Du et al., 2020). This study investigates the blood flow redistribution characteristics of healthy subjects and patients with neurological disorders under EECP stimulation to assess the variations in WSS-related factors such as AWSS, oscillatory shear index (OSI), and relative resident time (RRT).

## Clinical Experiment

### Subjects and Ethical Approval

Ten subjects were enrolled in the study, namely, five young and healthy volunteers as controls (three men and two women, 24.6 ± 2.7 years of age, 171.4 ± 5 cm in height, 55.4 ± 2.9 kg in weight) and five patients with neurological disorders (three men and two women, 63.4 ± 8.1 years of age, 163 ± 6.6 cm in height, 68 ± 5.4 kg in weight). All the patients suffered from chronic headaches and asked for medical treatment in the Departments of Neurology or Cardiology of the Eighth Affiliated Hospital of Sun Yat-sen University (SYSU). Color Doppler https://www.wisegeek.com/what-is-an-ultrasound.htm scans showed no significant carotid atherosclerotic plaques in any of the subjects. No severe cerebrovascular disease was diagnosed in any of the subjects. All the participants were asked to not consume nicotine, caffeine, or alcohol for at least 24 h before testing. A written informed consent was obtained from all the patients with CAD and controls before the experiment. This study was approved by the local medical ethics committee of the Eighth Affiliated Hospital of Sun Yat-sen University (SYSU).

### Medical Image Acquisition and Processing

All the subjects underwent the clinical protocol for carotid artery MRI on a 3.0 T MRI device (MAGNETOM Prisma, Siemens Electric Company, Munich, Germany). A two-part bilateral eight-channel carotid surface coil (XDR02021, Shenzhen Xindery Electronic Technology Co., Ltd., Shenzhen, China) was used for image acquisition.

### EECP Intervention Protocol and Color Doppler Ultrasound Measurement

All the subjects received a single 30-min session of EECP treatment with incremental external cuff pressures ranging from 20 to 40 kPa. A portable EECP device (P-ECP/TM, PSK-Health Sci-Tech Development Co., Ltd., Chongqing, China) was used. A Doppler flow velocity spectrum examination was performed in the right CCA, right ICA, and right ECA, 2–3 cm proximal to the bifurcation of the vessels, using a color Doppler ultrasound system (EPIQ 7, Philips, Eindhoven, The Netherlands) equipped with a 9–11 MHz multifrequency high-resolution linear probe. EECP treatment at all cuff pressures should continue for at least 5 min to ensure sufficient intervention; measurement should begin only when the finger pulse shown on the EECP device monitor has stable waveforms in continuous cardiac cycles.

**Figure 1:**
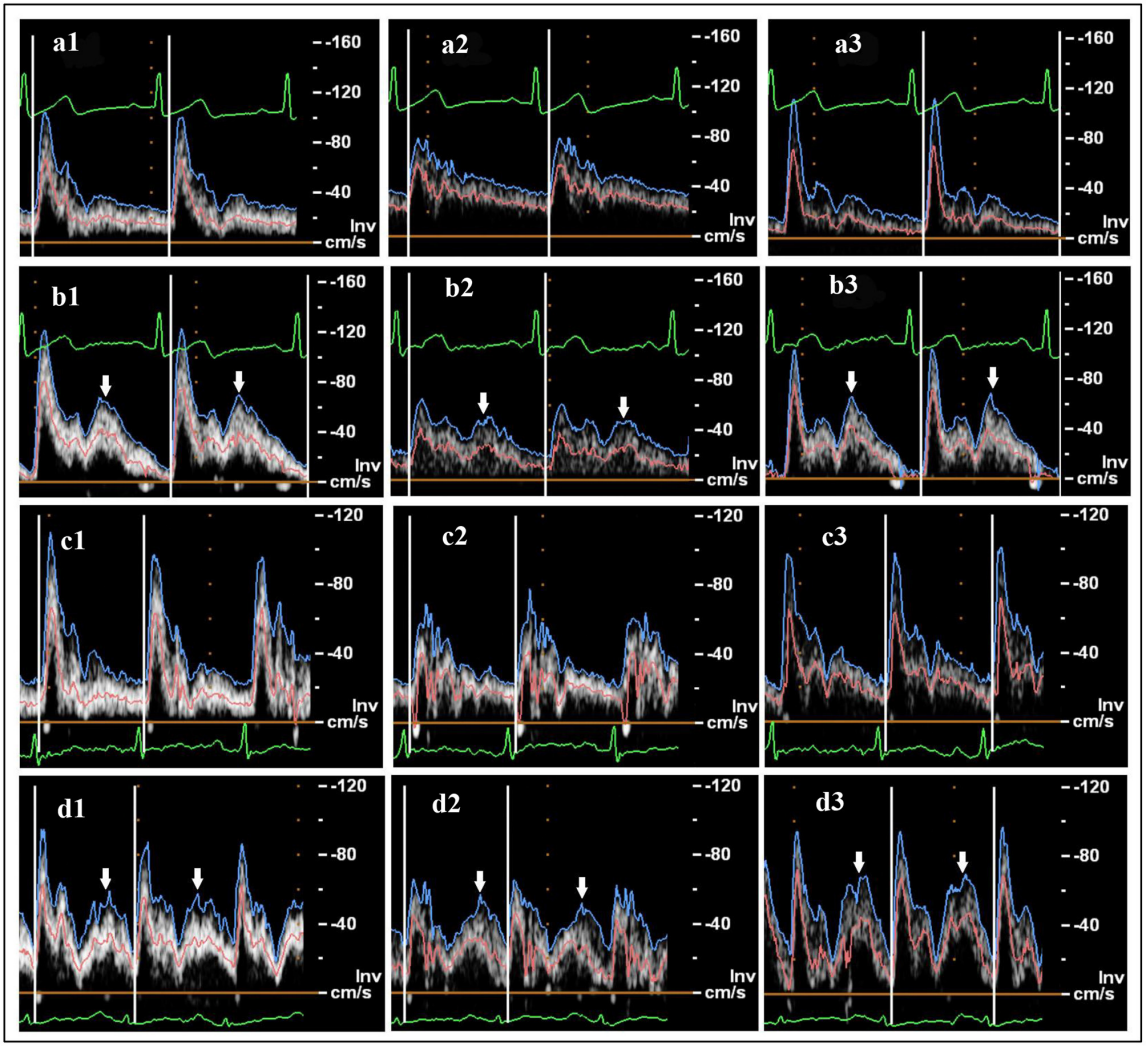
Ultrasound flow velocity spectrum: **(a)** healthy subject, pre-enhanced external counterpulsation (EECP); **(b)** healthy subject during EECP with 30 kPa cuff pressure; **(c)** patient, pre-EECP; **(d)** patient during EECP with 30 kPa cuff pressure. Common carotid artery (CCA), internal carotid artery (ICA), and external carotid artery (ECA) are represented by **1, 2**, and **3**, respectively. The blue, pink, and green curves represent the peak flow velocity waveforms, mean flow velocity waveforms, and ECGs, respectively. White arrows indicate the diastolic increase induced by EECP stimulation.

In this study, mean blood velocity waveforms were extracted from the ultrasound spectra (Figure 1, pink curves) for analysis and were used as boundary conditions for the numerical simulation. The mean lumen diameter of the vessel over one cardiac cycle was used to calculate the blood flow rate and volume, defined as:

Mean diameter = [(systolic diameter × 1/3)] + [(diastolic diameter × 2/3)] (Sato et al., 2011).

## Numerical Simulation Procedure and Boundary Conditions

### Geometry Reconstruction and Boundary Conditions

**Figure 2:**
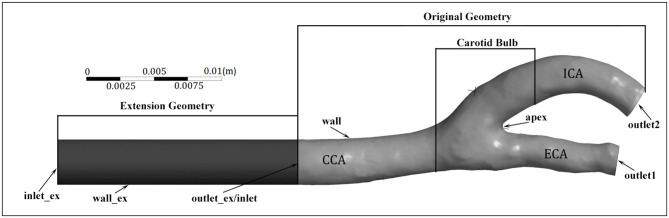
Geometry and boundaries of a carotid artery model. Note that the extension of the internal carotid artery (ICA) outlet is not included.

Geometry reconstruction based on MRI images was performed using MIMICS 17.0 (Materialise NV, Leuven, Belgium). The CFD model consisted of four boundaries, an inlet at the CCA, a wall, and outlets at the ICA and ECA (Figure 2). The blood flow rate curves were determined based on velocity waveforms measured *via* Doppler ultrasound, and served as the flow rate boundary conditions at the ICA and CCA. To achieve a fully developed flow, the inlet and outlets were extended outward along their normal direction for a length of five times their diameter. The pressure opening boundary condition was set at the ECA, and the arterial wall was assumed to be rigid and no-slip.

### Mesh Generation

Mesh generation was conducted based on the reconstructed model using ICEM 17.0 (Ansys Inc., Canonsburg, PA, United States). The geometry was meshed with tetrahedral cells in the core region. As the no-slip wall condition was used at the vessel wall, to improve solution accuracy, eight inflation layers were generated near the wall where a high variable gradient is present. A mesh-independence study was performed to obtain an optimized mesh producing reliable results and acceptable computational burdens.

### Governing Equations and the Solver

The blood fluid was assumed to be impressible and isoviscous (Newtonian type, μ = 0.039 Pa·s). Thus, the governing transport equations are the continuity and momentum equations, which can be expressed in the general form as

(1) $$\begin{array}{l} Continuity:\nabla U=0 \end{array}$$

(2) $$\begin{array}{l} Momentum:\rho \frac{DU}{Dt}=-\nabla p+\nabla \mu \overset{∙}{\gamma } \end{array}$$

where *p* is fluid pressure, ***g*** is gravitational acceleration, ***U*** is the velocity field, and $\overset{∙}{\gamma }$ is the second invariant of the shear rate tensor, defined as $\overset{∙}{\gamma }\equiv {\left[\frac{1}{2}\;\left(\overset{∙}{\gamma }:\overset{∙}{\gamma }\right)\;\right]}^{\frac{1}{2}}$.

The simulations were conducted using the Ansys Workbench commercial software. To discretize the governing Equations (1) and (2), a high-resolution advection scheme was implemented. The blend factor β was computed locally to be as close to 1 as possible, intending to satisfy the accuracy and boundedness requirements. The transient scheme used for the solution to march in time was the second-order backward Euler scheme. Unknowns were solved using this configuration for two cardiac cycles. The results of the first cardiac cycle were used to initialize the computational domain. The results presented in the following sections were produced from the second cardiac cycle. Numerical solutions were obtained; the root mean square (RMS) mass and momentum residuals were below 10^−5^. Achieving this level of convergence typically requires 20–40 iterations.

### Statistics

All statistical analyses were conducted using the SPASS 25.0 software. Data are presented as mean ± standard deviation. Statistical significance was set at *p* < 0.05.

## Results and Discussion

### Influence of EECP on Carotid Artery Lumen Diameter and Blood Flow

**Figure 3:**
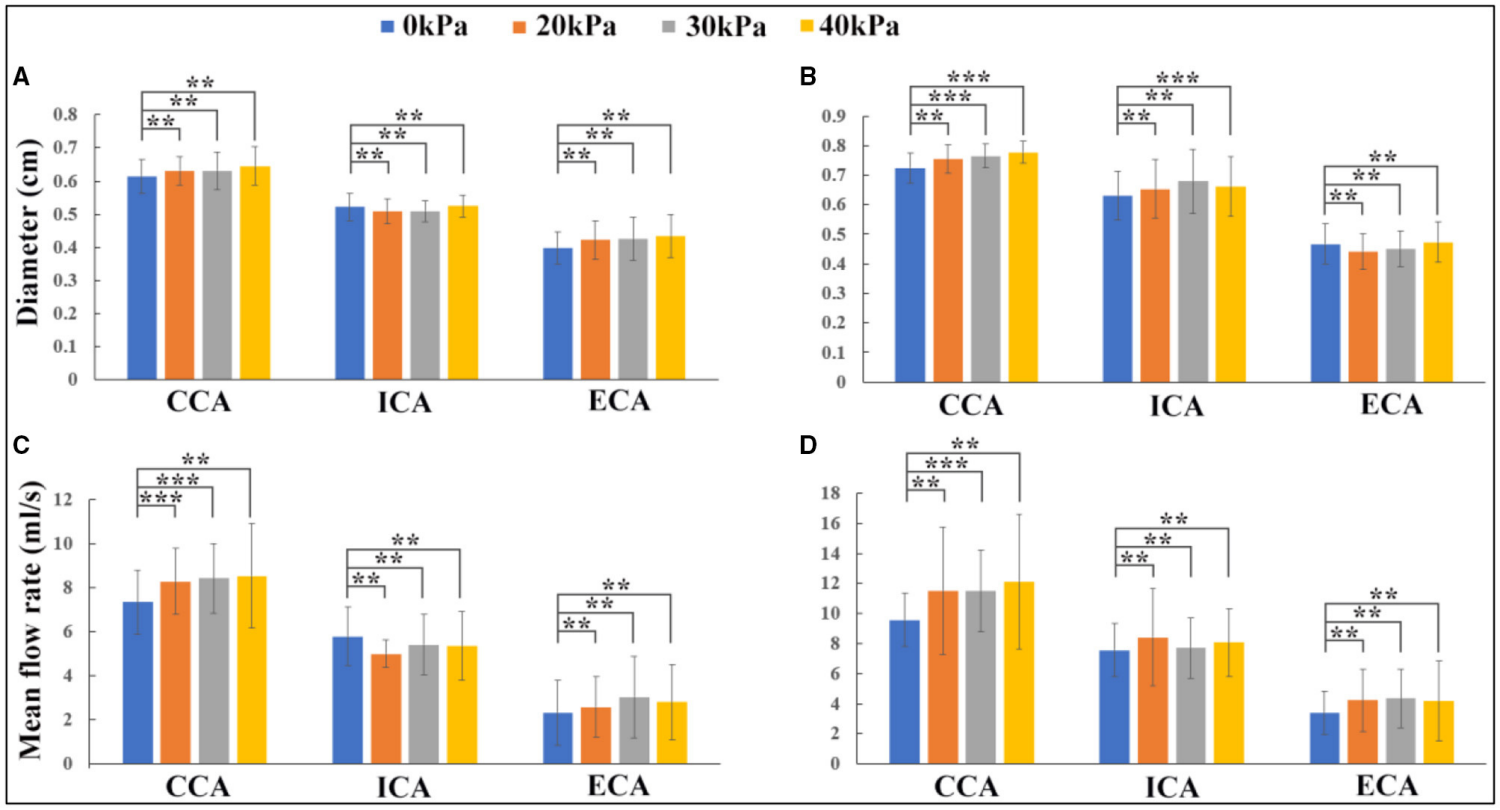
Effects of enhanced external counterpulsation (EECP) on lumen diameter and blood flow rate in carotid artery: **(A)** lumen diameter variations in the healthy control group; **(B)** lumen diameter variations in the patient group; **(C)** blood flow rate variations in the healthy control group; **(D)** blood flow rate variations in the patient group (**indicates *P* > 0.05, ***indicates *P* ≤ 0.05).

Variations in the carotid artery lumen diameter and blood flow rate before and during EECP stimulation with incremental external cuff pressures were calculated based on the Doppler ultrasound measurement, as shown in Figure 3. From statistical analysis, CCA and ECA diameters slightly increased during EECP compared with the pre-EECP condition in the healthy control group, although the increase was not statistically significant (*p* > 0.05). The ICA diameter in the healthy control group remained approximately constant. In the patient group, the CCA diameter significantly increased during EECP with cuff pressures of 30 and 40 kPa (*p* = 0.041 and *p* = 0.044, respectively). The ICA diameter increased during EECP compared with the pre-EECP condition, but only with a cuff pressure of 40 kPa; the increase was statistically significant (*p* = 0.036). The ECA diameter in the patient group remained approximately constant. For the healthy control group, EECP stimulation with cuff pressures of 20 and 30 kPa led to statistically significant increases in mean blood flow rate (MBFR) over a cardiac cycle in the CCA compared with the pre-EECP condition [7.339 ± 1.547 vs. 8.282 ± 1.497 ml/s (*p* = 0.034), and 7.339 ± 1.547 vs. 8.426 ± 1.595 ml/s (*p* = 0.017), respectively]. The MBFR in the ECA increased during EECP compared with the pre-EECP condition, although the increase was not statistically significant. The MBFR in the ICA during EECP showed slight decreases with cuff pressures of 20, 30, and 40 kPa compared with the pre-EECP condition (5.789 ± 1.327 vs. 4.991 ± 0.627 vs. 5.422 ± 1.362 vs. 5.356 ± 1.574 ml/s) but were not statistically significant (*p* > 0.05). The measurements support findings in previous studies (Jungehuelsing et al., 2010; Lin et al., 2014) and indicate that although EECP stimulation significantly elevated the CCA blood flow in the healthy subjects, the ICA blood flow level remained constant or slightly decreased. Enhanced blood flow in the CCA was transmitted to the ECA and led to a large increase in the ECA blood flow.

In the patient group, EECP stimulation led to MBFR increases in the CCA, ICA, and ECA, but in the CCA only when the EECP cuff pressure was 30 kPa; the MBFR increase was statistically significant compared with the pre-EECP condition (9.56 ± 1.768 vs. 11.485 ± 2.705 ml/s, *p* = 0.05). EECP stimulation with 20, 30, and 40 kPa cuff pressures led to 11.26, 1.84, and 6.92% MBFR increases in the ICA, respectively, compared with the pre-EECP condition. The results indicated that the influence of EECP on ICA blood flow was different in the patients than in the healthy subjects. The ICA blood flow in the patient group increased during EECP stimulation, resulting in an increase in cerebral blood flow, such as MCA blood flow (Lin W. H. et al., 2012; Lin et al., 2014). In this study, a rather low cuff pressure of 20 kPa (150 mmHg) produced the greatest blood flow increase in the ICA.

### Influence of EECP on WSS-Related Factors

Enhanced external counterpulsation induced significantly different blood flow redistributions in the carotid bifurcation in the healthy subjects and patients, which could lead to different biomechanical responses. Biomechanical forces, especially WSS, are widely believed to play an important role in maintaining the physiological functions of blood vessels (Brown et al., 2016). WSS-derived hemodynamic factors, such as AWSS, OSI, and RRT, are believed to quantify the levels and fluctuations of WSS over cardiac cycles, defined as (Rikhtegar et al., 2012).

(3) $$\begin{array}{l} AWSS=\;\frac{1}{T}\int_{0}^{T}\left|{\vec{\tau }}_{w}\right|dt \end{array}$$

(4) $$\begin{array}{l} OSI=\frac{1}{2}(1-\frac{\left|\int_{0}^{T}{\vec{\tau }}_{w}dt\right|}{\int_{0}^{T}\left|{\vec{\tau }}_{w}\right|dt}) \end{array}$$

(5) $$\begin{array}{l} RRT=\frac{1}{\left(1-2OSI\right)\times AWSS} \end{array}$$

Where $\left|{\vec{\tau }}_{w}\right|$ is the magnitude of the instantaneous WSS vector ${\vec{\tau }}_{w}$ and *T* is the duration of one cardiac cycle.

**Figure 4:**
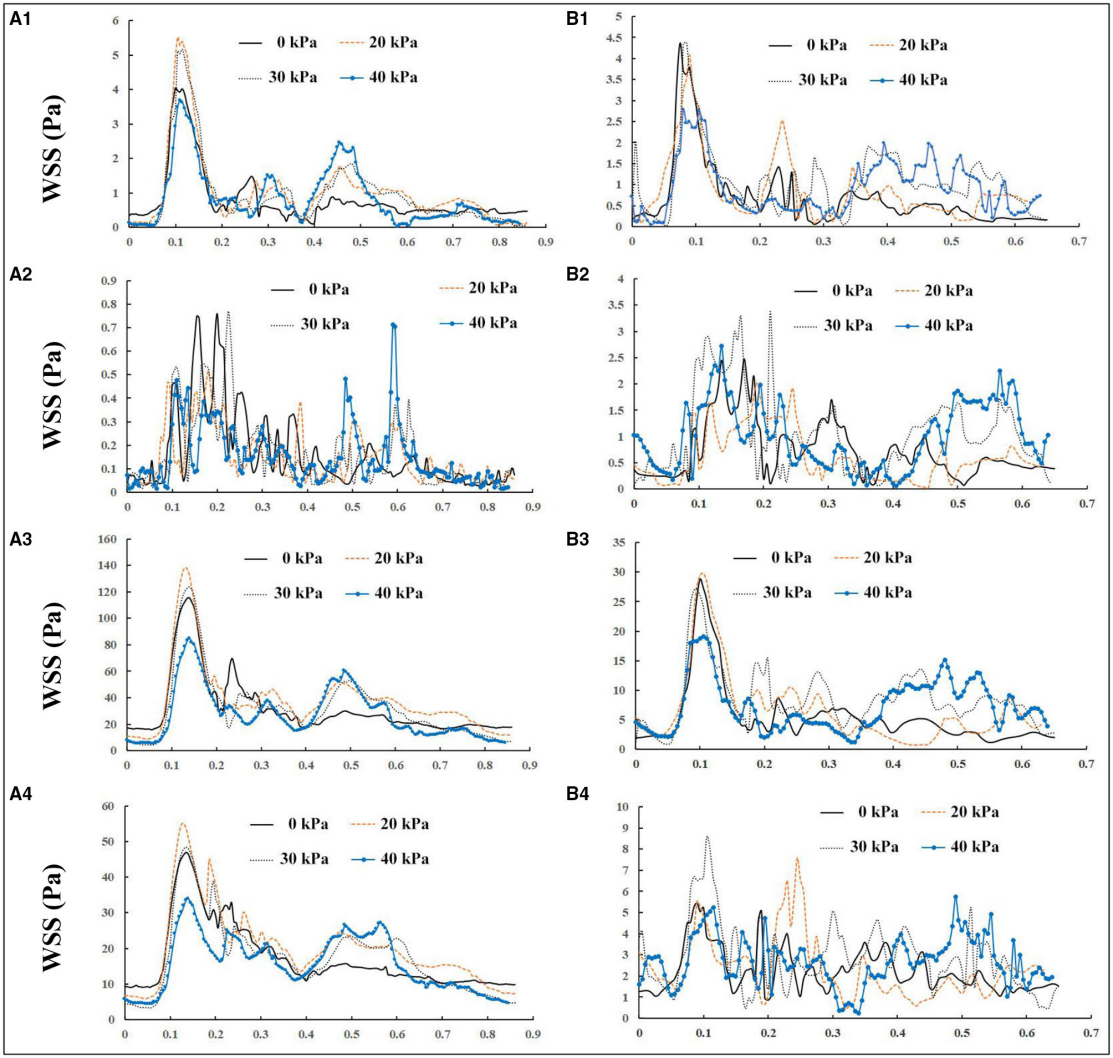
Wall shear stress (WSS) variations in one cardiac cycle before and during enhanced external counterpulsation (EECP) with incremental cuff pressures: **(A, B)** represent a healthy subject and a patient, respectively; **1, 2, 3**, and **4** represent the proximal common carotid artery (CCA), outer wall of the internal carotid artery (ICA) bulb, apex, and inner wall of proximal external carotid artery (ECA), respectively.

The wall shear stress exerted by blood flow is the tangential frictional force on the endothelial surface. It is believed that the initiation of atherosclerotic lesions is highly correlated with local areas of low WSS, defined as <1 Pa (Samady et al., 2011). High WSS up to a certain threshold seems to protect against plaque formation (Rikhtegar et al., 2012; Brown et al., 2016; Thondapu et al., 2017). WSS is largely dependent on the geometric characteristics of the vessel, such as curvature and diameter. The WSS distributions of a healthy subject and a patient at four different points in the carotid bifurcation wall (proximal CCA, outer wall of the ICA bulb, inner wall of the proximal ECA, and apex) before and during EECP intervention with incremental pressures are shown in Figure 4. In both subjects, WSS distributions were much more complicated at the ICA bulb than at other sites. EECP mainly influenced the WSS distribution in the diastole, especially at the proximal CCA and apex. Peak WSS values were observed at the apex of the bifurcation (116.78 Pa before EECP vs. 138.28 Pa during EECP for the healthy subject, and 29.33 Pa before EECP vs. 29.78 Pa during EECP for the patient subject).

**Figure 5:**
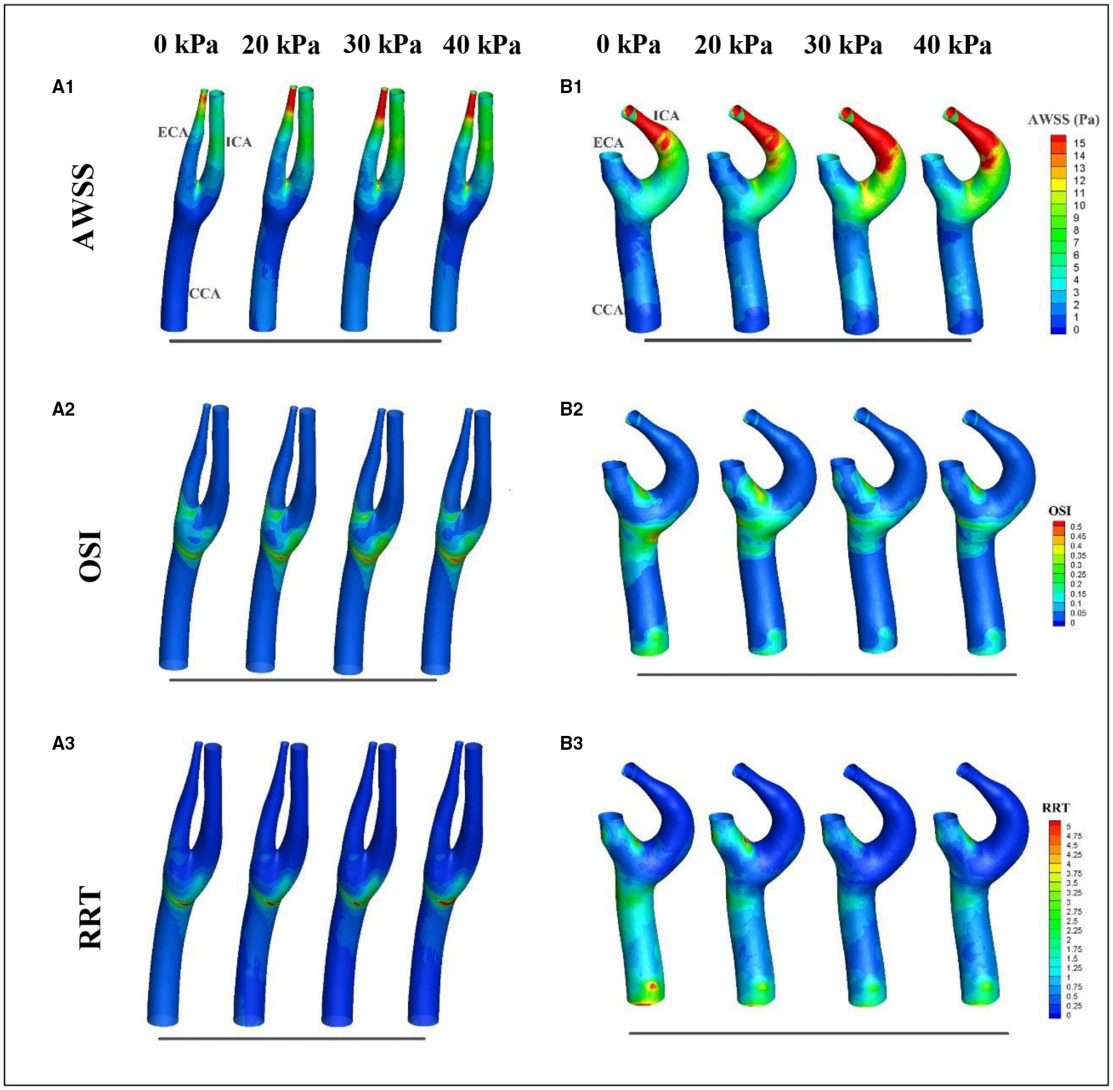
Variations in wall shear stress (WSS)-derived hemodynamic factors over the cardiac cycle before and during EECP: **(A,B)** represent the healthy subject and the patient, respectively; **1, 2**, and **3** represent averaged wall shear stress (AWSS), oscillatory shear index (OSI), and relative resident time (RRT), respectively.

**Figure 6:**
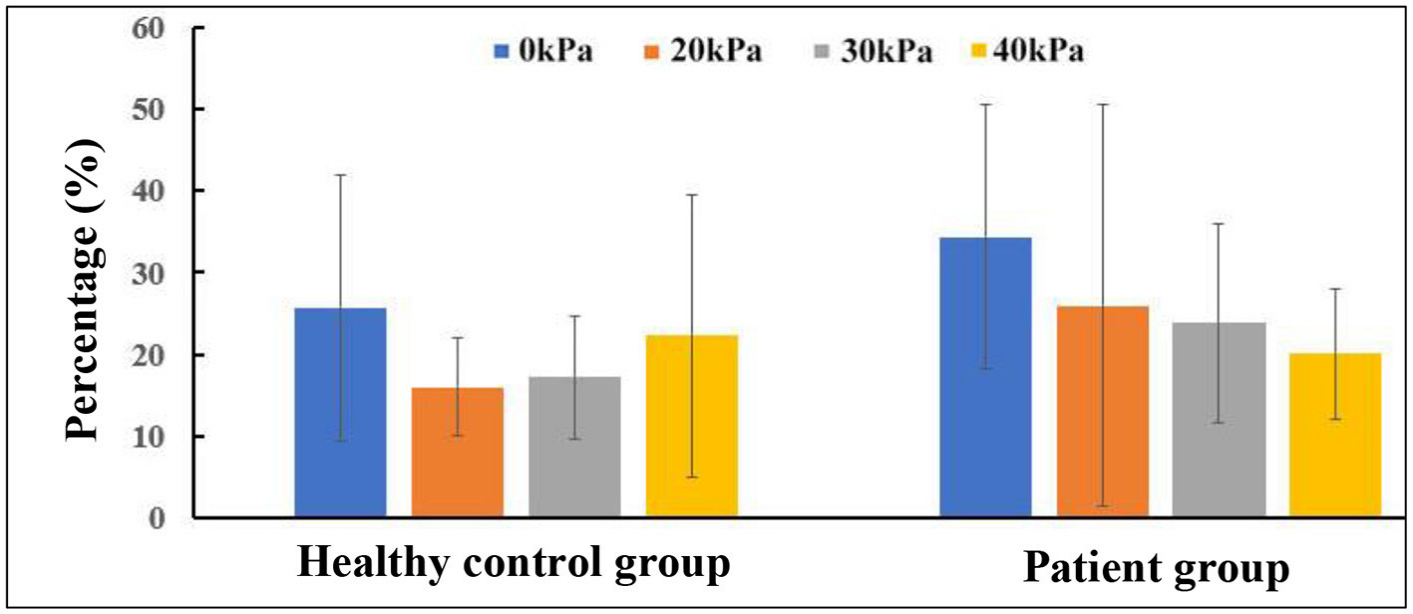
Changes in percentage of low-averaged wall shear stress (AWSS) segments in carotid bifurcation.

Considering that WSS is a continuous and oscillatory hemodynamic parameter in cardiac cycles, AWSS represents the WSS magnitude averaged over the cardiac cycle, and has been confirmed to be highly sensitive in predicting plaque locations (Rikhtegar et al., 2012). Samady et al. (2011) indicated that an increase in the atherosclerotic plaque area was associated with low-AWSS (<1 Pa) arterial segments, and a decrease in plaque area was correlated with intermediate-AWSS (≥1 and <2.5 Pa) and high-AWSS (≥2.5 Pa) arterial segments. The AWSS contours for a healthy subject and a patient are shown in Figures 5A1,B1; the influence of EECP on WSS distribution is significantly different between the subjects, mainly because of different blood flow redistribution characteristics. For the healthy subject, low-level AWSS segments appeared at the proximal CCA and outer wall of the carotid bulb, consistent with the results reported by Li et al. (2018). EECP stimulation influenced the AWSS mainly at the apex and the ECA. However, for the patient subject, EECP influenced the AWSS over the entire carotid artery bifurcation, especially at the ICA. The percentages of low-AWSS (<1Pa) arterial segments over the carotid bifurcations were calculated and compared before and during EECP with incremental cuff pressures (Figure 6). The percentage of the low-AWSS segments decreased during EECP for the healthy control group and the patient group compared with the pre-EECP condition. However, no statistically significant difference was found in any of the cases (*p* > 0.05).

Oscillatory shear index is a factor believed to quantify the temporal oscillation of the WSS. Some studies have reported a positive correlation between high levels of OSI and plaque formation (Rikhtegar et al., 2012). Figures 5A2,B2 show the OSI contours of a healthy subject and a patient, before and during EECP with incremental cuff pressures. The results indicate that high-OSI segments appear at the proximal CCA, bulb, and inner wall of the proximal ECA in the healthy subject and the patient. However, the influence of EECP on OSI was significantly different for the two subjects. For the healthy subject, EECP increased the OSI at the bulb, which was approximately proportional to the external cuff pressure; the OSI at the ECA remained constant. For the patient subject, the OSI at the bulb and proximal CCA decreased during EECP compared with the pre-EECP condition, and generally showed a negative correlation with the cuff pressure. The OSI at the proximal ECA was constant or slightly increased during EECP compared with the pre-EECP condition.

Figures 5A3,B3 show the variations in the RRT distributions before and during EECP with incremental cuff pressures. The RRT is a factor introduced to quantify the residence time of molecules in arterial segments; its value is determined by both AWSS and OSI. Some studies have reported a positive correlation between sites of plaque initiation and high RRT (Hoi et al., 2011; Rikhtegar et al., 2012). The results of this study indicated that for the healthy subject, a high-RRT segment appeared at the bulb and proximal CCA, and EECP led to a plateau or a slight increase in the RRT over the carotid bifurcation. However, for the patient subject, high-RRT segments appeared at the distal and proximal CCA and the inner wall of the ECA. The RRT decreased in the CCA during EECP compared with the pre-EECP condition; no significant difference was observed with different cuff pressures. The RRT increased in the ECA during EECP with a relatively low cuff pressure of 20 kPa compared with the pre-EECP condition. However, with relatively high cuff pressures of 30 and 40 kPa, the RRT in the ECA decreased.

The local distribution characteristics of WSS-derived hemodynamic factors such as the AWSS, OSI, and RRT are strongly dependent on the patient-specific geometric characteristics of the vessel; these factors may be significantly different between subjects (Glor et al., 2003). Considering that the carotid bifurcation is a high-risk atherosclerotic lesion region, the focus of this study was the influence of EECP intervention on variations in WSS-derived hemodynamic factors over the local bifurcation region including the proximal CCA, ICA bulk, and proximal ECA. Table 1 shows the variations in WSS-derived hemodynamic factors before and during EECP. The statistical results indicate that EECP induced an increase in AWSS in both groups. For the healthy control group, a relatively low cuff pressure of 20 kPa produced a maximum AWSS increase of 19.83% compared with the pre-EECP state. For the patient group, the optimal cuff pressure for an increase in WSS was 30 kPa, which produced a 38.5% increase in AWSS compared with the pre-EECP state. The OSI decreased 16.28% during EECP with a cuff pressure of 20 kPa, and increased 16.28 and 39.53% during EECP with 30 and 40 kPa cuff pressures, respectively, compared with the pre-EECP state. For the patient group, the OSI increased 8.33 and 3.33% during EECP with 20 and 30 kPa cuff pressures, respectively, and increased 11.67% during EECP with a cuff pressure of 40 kPa compared with the pre-EECP state. The RRT decreased in both groups during EECP compared with the pre-EECP state. For the healthy control group, a relatively low cuff pressure of 20 kPa produced a 22.69% decrease in the maximum RRT compared with the pre-EECP state. For the patient group, a negative correlation was observed between the RRT and the EECP cuff pressure. Compared with the pre-EECP state, the RRT decreased by 6.18, 19.66, and 22.47% during EECP with cuff pressures of 20, 30, and 40 kPa, respectively. Because of the relatively small sample size in this study and the significant difference in the WSS-derived hemodynamic factors at the same sites in different subjects, no statistically significant difference was found. However, the results indicate consistent variation characteristics induced by EECP with incremental cuff pressures.

**Table 1:** Variations in wall shear stress (WSS)-derived hemodynamic factors before and during enhanced external counterpulsation (EECP).

	**Pre-EECP**		**20 kPa EECP**		**30 kPa EECP**		**40 kPa**	
	**HC**	**P**	**HC**	**P**	**HC**	**P**	**HC**	**P**
AWSS (Pa)	4.79 ± 2.73	2.26 ± 1.49	5.74 ± 2.47	2.95 ± 1.91	5.28 ± 1.89	3.13 ± 2.61	5.12 ± 1.49	3.01 ± 2.12
OSI	0.043 ± 0.017	0.06 ± 0.021	0.036 ± 0.011	0.055 ± 0.018	0.05 ± 0.013	0.058 ± 0.02	0.06 ± 0.022	0.067 ± 0.02
RRT	1.19 ± 0.19	1.78 ± 0.84	0.92 ± 0.16	1.67 ± 1.08	1.05 ± 0.12	1.43 ± 0.62	0.99 ± 0.27	1.38 ± 0.62

*HC indicates the healthy control group (n = 5); P indicates the patient group (n = 5). Data represent mean ± SD. For all the three factors, no statistically significant difference (p > 0.05) was found between the pre-EECP state and EECP states, or between EECP states with different cuff pressures*.

## Limitations

As numerical simulation and subsequent post-processing are time-consuming, and considering the exploratory nature of this study, the sample size was relatively small. This is probably the main reason why the statistical results indicate no statistical significance in most cases. Properly powered studies are necessary in the future to confirm these relationships. Another limitation is that the Doppler ultrasound measurements were not isochronous in the CCA, ICA, and ECA, because two or more probes were not available at the right or left carotid bifurcation. Furthermore, in this study, autoregulatory index (ARI) evaluations of the patients to verify an impaired CA were not performed.

## Conclusion

To the best knowledge of the authors, this is the first prospective investigation of the influence of EECP treatment on blood flow distribution and WSS-derived hemodynamic factors in the carotid bifurcation. This study indicated that EECP intervention increased ICA blood flow and WSS in the carotid bifurcation in patients with neurological disorders, which may be the most important hemodynamic mechanism underlying the clinical benefits of EECP, such as improvement of cerebral ischemia and vascular endothelial function. However, in the healthy subjects, the ICA blood flow remained constant or slightly decreased during EECP treatment, although the CCA blood flow significantly increased, avoiding the potential risk of cerebral hyperperfusion *via* the ICA. The physiological phenomenon induced by EECP is similar to moderate- and high-intensity dynamic physical exercise (Sato et al., 2011). CA and impaired CA may play a key role in modulating carotid hemodynamics in both physical exercise and EECP, but the mechanism requires further investigation.

To achieve a better cerebral blood flow increase *via* the ICA, a relatively low external cuff pressure of 20 kPa is recommended as the optimal EECP treatment pressure for cerebrovascular diseases, and this can also reduce the potential risk of adverse events associated with higher pressures (Lin et al., 2014). Moreover, EECP treatment with a cuff pressure of 20 kPa can induce an increase in the WSS in the carotid bifurcation, and a decrease in temporary WSS oscillation in healthy subjects and patients with neurological disorders.

## Data Availability Statement

The raw data supporting the conclusions of this article will be made available by the authors, without undue reservation.

## Ethics Statement

The studies involving human participants were reviewed and approved by the local medical ethics committee of the Eighth Affiliated Hospital of Sun Yat-sen University (SYSU). The patients/participants provided their written informed consent to participate in this study.

## Author Contributions

ST conducted the numerical simulation. WP designed the clinical measurement scheme and participated in the measurements. JP and YW performed the MRI test. BD and YL participated in the measurements and analyzed the ultrasound data. HW performed the ultrasound measurement. XL reconstructed the arterial model. BL designed the clinical measurement and provided the patient subjects. JD designed and sponsored this study, participated in the measurements, and wrote the initial draft. All authors contributed to the article and approved the submitted version.

## Conflict of Interest

The authors declare that the research was conducted in the absence of any commercial or financial relationships that could be construed as a potential conflict of interest.

## Publisher's Note

All claims expressed in this article are solely those of the authors and do not necessarily represent those of their affiliated organizations, or those of the publisher, the editors and the reviewers. Any product that may be evaluated in this article, or claim that may be made by its manufacturer, is not guaranteed or endorsed by the publisher.
